# Efficient Non-Viral Gene Modification of Mesenchymal Stromal Cells from Umbilical Cord Wharton’s Jelly with Polyethylenimine

**DOI:** 10.3390/pharmaceutics12090896

**Published:** 2020-09-22

**Authors:** Ana Isabel Ramos-Murillo, Elizabeth Rodríguez, Karl Beltrán, Cristian Ricaurte, Bernardo Camacho, Gustavo Salguero, Rubén Darío Godoy-Silva

**Affiliations:** 1 Chemical and Biochemical Processes Research Group, Department of Chemical and Environmental Engineering, Faculty of Engineering, Universidad Nacional de Colombia, Bogotá D.C. 111321, Colombia; airamosmu@unal.edu.co (A.I.R.-M.); elrodriguezca@unal.edu.co (E.R.); 2 Advanced Therapies Unit, Instituto Distrital de Ciencia, Biotecnología e Innovación en Salud (IDCBIS), Bogotá D.C. 111611, Colombia; kbeltran@idcbis.org.co (K.B.); caricaurte@idcbis.org.co (C.R.); bacamacho@idcbis.org.co (B.C.); gsalguero@idcbis.org.co (G.S.)

**Keywords:** gene therapy, differentiation, cationic polymer, immunophenotype, immunomodulation, cell therapy, standardization, polyplexes

## Abstract

Mesenchymal stromal cells (MSC) derived from human umbilical cord Wharton’s jelly (WJ) have a wide therapeutic potential in cell therapy and tissue engineering because of their multipotential capacity, which can be reinforced through gene therapy in order to modulate specific responses. However, reported methodologies to transfect WJ-MSC using cationic polymers are scarce. Here, WJ-MSC were transfected using 25 kDa branched- polyethylenimine (PEI) and a DNA plasmid encoding GFP. PEI/plasmid complexes were characterized to establish the best transfection efficiencies with lowest toxicity. Expression of MSC-related cell surface markers was evaluated. Likewise, immunomodulatory activity and multipotential capacity of transfected WJ-MSC were assessed by CD2/CD3/CD28-activated peripheral blood mononuclear cells (PBMC) cocultures and osteogenic and adipogenic differentiation assays, respectively. An association between cell number, PEI and DNA content, and transfection efficiency was observed. The highest transfection efficiency (15.3 ± 8.6%) at the lowest toxicity was achieved using 2 ng/μL DNA and 3.6 ng/μL PEI with 45,000 WJ-MSC in a 24-well plate format (200 μL). Under these conditions, there was no significant difference between the expression of MSC-identity markers, inhibitory effect on CD3+ T lymphocytes proliferation and osteogenic/adipogenic differentiation ability of transfected WJ-MSC, as compared with non-transfected cells. These results suggest that the functional properties of WJ-MSC were not altered after optimized transfection with PEI.

## 1. Introduction

Mesenchymal stem cells (MSC) was the name proposed in 1991 by Arnold Caplan for a class of cells isolated from human and mammalian periosteum and bone marrow, able to be expanded in culture while preserving their in vitro capacity to form several mesodermal phenotypes and tissues. Additionally, they were proposed as multipotent cells able to proliferate and differentiate into several lineages depending on the environmental conditions [1]. Due to the heterogeneity of isolation and cultivation procedures among laboratories, in 2006, the International Society for Cellular Therapy (ISCT) proposed that the abbreviation MSC should be used to designate multipotent mesenchymal stromal cells and established a position statement about the minimal criteria to call them this way. These criteria were adherence to plastic in standard culture conditions, specific surface antigen (Ag) expression, positive (≥95%) for CD73, CD90 and CD105 and negative (≤2%) for CD12B or CD14, CD34, CD45, CD79α or CD19 and HLA-DR, as measured by flow cytometry and multipotent differentiation potential (osteoblasts, adipocytes and chondroblasts) demonstrated by staining of in vitro cell culture [2].

Bone marrow (BM) has been the most common source of MSC [3,4,5]; however, their clinical application is limited because their collection is invasive, painful [6], and has low cell yield [7]. As a result, new sources of MSC have been explored. The umbilical cord (UC) is a conduit between the placenta and fetus, and it is surrounded by a gelatinous substance made up of mucopolysaccharides (chondroitin sulfate and hyaluronic acid) named Wharton’s jelly (WJ) [8,9]. WJ has been characterized as a promising source of MSC since derived cells have some advantages over other sources of multipotent stromal cells [9,10]. WJ-MSC display higher proliferation, lower senescence rates, and relatively higher expression of pluripotency markers than stromal cells obtained from other sources [11,12,13,14]. Furthermore, since WJ-MSC are obtained from the neonatal umbilical cord, which is considered a biological waste [15], the ethical concerns are strongly reduced [10,11,14]. 

Several researchers have found that regenerative capacity in vivo of MSC-based therapies is sub-optimal due their low survival, poor biodistribution and reduced differentiation rates [16,17,18]. To overcome these difficulties, MSC can be modified via genetic engineering to improve their survival rate, increase the secretion of differentiation factors needed to induce a specific lineage [19], improve the immunomodulatory capacity and increase the secretion of cytokines [20], among others [21,22]. Additionally, the discovery of several genes related to the repair of damaged tissues brings the opportunity to genetically modified cells, such as MSC, with huge potential in the treatment of diseases that benefit from transitory or durable expression of therapeutic genes [21].

The success of gene delivery is highly dependent on the carrier employed to transfer DNA or RNA. Non-Viral vectors such as liposomes and cationic polymers are preferred when transient modifications are expected because they are safe and easily scalable, albeit their low transfection efficiency and short transgene half-life, thereby limit their clinical application [23]. In this regard, the use of polyethylenimine (PEI) as a transfection agent has emerged as a potential candidate for engineering MSC from different sources [24,25,26] displaying satisfactory results. PEI is a cationic polymer widely used for non-viral transfection because it combines strong DNA compaction with a potential for endosomal escape [27].

While there is a vast amount of literature regarding methodologies for PEI-driven transfection of MSC from diverse sources, to date, quite few reports exist on the use of cationic polymers in the transfection of umbilical cord MSC. Bahadur et al. (2015), conjugated 1.2 kDa PEI with linoleic acid and hyaluronic acid and evaluated transfection in MSC of bone marrow (BM) and umbilical cord (UC) and used 25 kDa branched PEI as transfection control [28]. Although they reached transfection efficiencies of up to 40% in UC-MSC, the viability was reduced to 60% or lower, making it a methodology that can be improved [29]. With the goal of transfecting UC-MSC with functionalized particles, Wang et al. (2017) included a modified HIV-1 trans-activator of transcription protein (TAT) into the transfecting particles; they reached a peak transfection efficiency of 12% with 25 kDa branched PEI vs. 14% with the functionalized particles, although they found significant differences in cell viability with both approaches, going from 70 to 90%, respectively [29]. Unfortunately, both mentioned papers evaluated the expression of surface markers to confirm the identity of MSC before transfection but there is no indication in their work related to the effect of transfection on the expression of those markers, which is essential for further use of transfected MSC in clinical applications.

The main aim of this work was to evaluate the non-viral gene modification of WJ-MSC by using PEI. We evaluated a methodology that combined optimal PEI ratios using smaller amounts of DNA to increase transfection efficiencies. Additionally, we were able to confirm that major biological properties of WJ-MSC including cell viability, immunophenotype, immunomodulatory capacity and expression of MSC-markers were preserved after PEI transfection, favoring the use of PEI as the optimal methodology for transient gene of WJ-MSC for future clinical translation.

## 2. Materials and Method

### 2.1. Subsection

Cell culture: Growth media Dulbecco’s Modified Eagle’s medium (DMEM)11885-084, Gibco, Life Technologies Corp., Carlsbad, CA, USA) and fetal bovine serum FBS (12103C, Sigma Aldrich, St. Louis, MO, USA) were employed for WJ-MSC culture. Roswell Park Memorial Institute (RPMI) 1640 medium (61870-036, Gibco, Life Technologies Corp., Carlsbad, CA, USA) was used to culture peripheral blood mononuclear cells (PBMC). Transfection: 25 kDa branched polyethylenimine (PEI) (408727, Sigma Aldrich, St. Louis, MO, USA) was employed in all transfection assays. Nucleus staining DAPI (4′,6-diamidino-2-phenylindole) was used at 300 nM (D9542, Sigma Aldrich, St. Louis, MO, USA) in phosphate-buffered solution 1X (PBS) at pH 7.4. Cell viability: Resazurin sodium salt (121519, PanReac AppliChem, Darmstadt, Germany) in PBS at pH 7.4 was used to evaluate the metabolic activity of cells as a measurement of cell viability. *Escherichia coli* DH5-α culture: Luria Bertani (LB) broth, Miller (Ref. L3152), and ampicillin sodium salt (Ref. A0166) were acquired from Sigma Aldrich (St. Louis, MO, USA) and agar-agar (A0949) was supplied by PanReac AppliChem (Darmstadt, Germany).

### 2.2. Expansion of WJ-MSC

WJ-MSC were obtained from umbilical cord donors (donors 40 and 148) from the Advanced Therapies Unit at the IDCBIS, following informed consent from mothers. WJ-MSC at passages 1 to 6 were cultured in low-glucose DMEM supplemented with 10% FBS at 37 °C and 5% CO_2_.

### 2.3. Plasmid Propagation

Competent *E. coli* DH5-α cells (kindly donated by Dr. Velásquez laboratory at Universidad Nacional de Colombia) were transformed with a DNA plasmid (RRL.SIN.cPPT-hCMV-eGFP) coding for GFP and resistance to ampicillin. Transformed *E. coli* cells were cultured in Luria Bertani (LB) medium and LB with agar-agar and selected with ampicillin. The GFP plasmid (DNA) was purified using a ZymoPURE™ plasmid maxiprep kit (catalog #D4203, Zymo Research, Irvine, CA, USA) following the recommended manufacturer procedure. The DNA concentration and purity were measured using a NanoDrop™ 2000/2000c Spectrophotometer (ThermoScientific^®^, Wilmington, DE, USA).

### 2.4. PEI and DNA Nanoparticle Formulations and Physicochemical Characterization

Branched PEI (25 kDa) was employed as the transfection agent. A PEI stock solution at 10 µg/µL was prepared in distilled water (DW), and the pH was adjusted to 3.15 with hydrochloric acid (HCl) (H1758, Sigma Aldrich, St. Louis, MO, USA). The PEI stock solution was filtered using a 0.2 µm filter. PEI working solutions at 1 µg/µL were prepared by dilution of the PEI stock solution in sterile DW and were aliquoted at 500 µL before freezing at −20 °C. In all experiments, fresh working PEI solutions were used to avoid repeating cycles of freezing and thawing. Transfection efficiency was evaluated at different N/P ratios (molar ratio of nitrogen in PEI/molar ratio of phosphorus in DNA). It was considered that every basic amino group of PEI was potentially responsible for DNA binding. One nitrogen (N) per repeat unit of PEI is CH_3_CH_2_NH*_x_*, where *x* is the average number of protons attached to N, (*x* = 2 × %NH_2_ (percentage of primary amines)+ 1 × %NH (percentage of secondary amines)). Thus, Mw: 43.1 g/mol [30,31]. At DNA, the repeating unit is a nucleotide, which has one phosphorus atom and an average molecular weight of 330 g/mol, approximately. As a rule, there are 3 nmol of P every µg of DNA, approximately [24,30,32,33]. In this work, the overall nitrogen content was quantified using a Total Organic Carbon/Total Nitrogen (TOC/TN) analyzer (Analytik Jena, Jena, Germany). Our analyses showed that a 50 mg/L solution of PEI in DW contained 14.6 mg/L of TN. According to the elemental composition of the repeating PEI unit, the theoretical TN should be 16.3 mg/L; thus, we adjusted our PEI content to 90% purity, considering that PEI is highly hygroscopic, and this difference corresponds to hydrated PEI. One of the main limitations of using PEI is the difficulty in obtaining reproducible results and comparing them with the results reported by other authors. To avoid these difficulties, Table 1 displays the detailed N/P ratio calculations used in this work.

According to Table 1, different N/P ratios were evaluated using 400 ng of DNA. DNA plasmid aliquots and PEI concentrations were adjusted to maintain the same volume of both solutions in every mixture. Transfection assays were carried out in 24-well plates. Each well had a superficial area of 2 cm^2^; thus, a minimum volume of 0.2 cm^3^ (200 µL) was required to cover all the surface. Several authors recommend avoiding exceeding 10% of the volume with complexes [30,34]. Therefore, considering that all the experiments were made in triplicate, the complexes were prepared in a final volume of 70 µL. This means that 35 µL of PEI solutions in DW at different concentrations were added to 35 µL DNA plasmid solution at 40 ng/µL, and the solution was vortexed immediately. After 30 min of incubation at 37 °C, the complexes were resuspended in 625 µL of prewarmed DMEM to obtain a final volume of 700 µL. Next, 200 µL of the complexes in DMEM were added to each well.

To evaluate the effect of the N/P ratio on the mean hydrodynamic diameter and zeta potential, several measurements were carried out using dynamic light scattering (DLS) and laser doppler micro-electrophoresis (LDME) (Zetasizer Nano ZS, Malvern Panalytical, Worcestershire, UK), respectively. This technique requires the dilution of the sample in 1 mL of distilled water doubly filtered (DWDF) (0.22 µm). In this case, 120 µL of complexes prepared in 4 batches of 30 µL (15 µL of DNA and 15 µL of PEI solution) were mixed with 1 mL of DWDF. The first measurements carried out were the mean hydrodynamic diameters. Then, the same sample was used to measure the Zeta potential. All measurements were carried out in triplicates. Complexes formed were also evaluated by electrophoresis in agarose gel, which contained 0.79 g of agarose and 100 mL of TAE buffer 0.5× stained with Ethidium Bromide. Bio-Rad power supply was employed to apply 100 V through the agarose gel for 30 min. 1 Kb Plus DNA Ladder (10787-018, Invitrogen, Vilnius, Lithuania) was used for sizing and quantification of double stranded DNA on agarose gels. BlueJuice™ (10816-015, Invitrogen, Vilnius, Lithuania) was used as loading buffer.

### 2.5. Growth Kinetics of WJ-MSC

To evaluate the effect of the initial cell concentration on the growth kinetics of WJ-MSC, two different seeding densities were evaluated: 2000 and 9000 WJ-MSC/cm^2^. WJ-MSC were cultured in a 48-well plate for 24 h, and then the medium was removed from the first 6 wells of the 48-well plate and was replaced with 100 µL of resazurin solution in supplemented DMEM at 44 µM. The whole plate was incubated at 37 °C and 5% CO_2_ in a Cytation^®^ 3 microplate reader (Biotek^®^, Winooski, VT, USA), and metabolic activity was monitored for 24 h by fluorescence. At this time, the same procedure was carried out for the next six different wells until day 6. In this way, the error due to the possible cytotoxic effect of resazurin on the cells is reduced.

### 2.6. Assessment of Polyethylenimine (PEI) Cytotoxicity on WJ-MSC

Metabolic activity was established as a measure of the variation of cell viability, through the reduction of resazurin to resorufin. PEI cytotoxicity was evaluated using the resazurin assay technique. For this purpose, 4.5 × 10^4^ WJ-MSC per well were cultured in a 24 well plate for 24 h. Next, culture medium was removed, and cells were treated with PEI and PEI/DNA complexes in DMEM without FBS at different concentrations for 4 h at 37 °C and 5% CO_2_. After that time, PEI or PEI/DNA solutions were removed, and 100 µL of resazurin solution 44 µM in DMEM + 10% FBS were added to each well and fluorescence intensity was quantified every hour using a Cytation 3 microplate reader with wavelengths of 530 and 590 nm for excitation and emission, respectively. All assays were conducted in triplicates. Cells treated with DMEM without FBS were used as controls.

### 2.7. In Vitro Evaluation of Transfection Efficiency of PEI–DNA Complexes in WJ-MSC

Samples of 1 × 10^4^ WJ-MSC/cm^2^ in DMEM + 10% FBS were cultured in 24-well plates for 48 h before transfection at 37 °C, 5% CO_2_. At this time, media were removed from wells and 20 μL of complexes of PEI and DNA (prepared as previously described) were resuspended in 180 μL of DMEM without FBS and the mixture was added to each well carefully. The plate was centrifuged at 280× *g* for 5 min and the cells were incubated for 4 h with the complexes. Then complexes were removed, replaced with DMEM + 10% FBS and cells were incubated again. After 48 h of transfection, WJ-MSC were trypsinized, recovered by centrifugation and resuspended in 100 μL of PBS 1X; GFP expression was evaluated by flow cytometry using a FACSCanto II (Becton Dickinson, San Jose, CA, USA). Data were analyzed using FlowJo™ Software V10.7.1 (Becton, Dickinson and Company, Ashland, OR, USA). Untreated cells and cells treated with non-complexed plasmid were used as controls. In every treatment, data are represented by the mean and standard deviation (SD) of three replicates.

### 2.8. Immunophenotype of WJ-MSC Transfected with PEI

To evaluate the presence of characteristic cell surface markers of MSC and the absence of hematopoietic cell markers, WJ-MSC were harvested by trypsinization, 48 h after the transfection protocol with PEI at an N/P ratio of 14 (720 ng PEI/90,000 cells). The expression of MSC-related cell surface antigens was assessed by flow cytometry using the membrane markers CD90 (APC), CD73 (PECy7), CD105 (PE), CD45 (APC/Cy7), CD34 (PerCP-Cy5.5), HLA-DR (Pacific Blue), and HLA-ABC (Clone W6/32, FITC). Cells were incubated for 30 min at 4 °C, then, centrifuged at 300× *g* for 6 min and resuspended in 0.2 mL PBS. Cell acquisition and MSC phenotyping was performed using a FACSCanto II flow cytometer and data were analyzed with FlowJo™ Software V10.7.1 (Becton, Dickinson and Company, Ashland, OR, USA). Non-Transfected WJ-MSC were used as the negative control.

### 2.9. Immunomodulation Potency Assay of PEI-Transfected WJ-MSC

**Figure 1:**
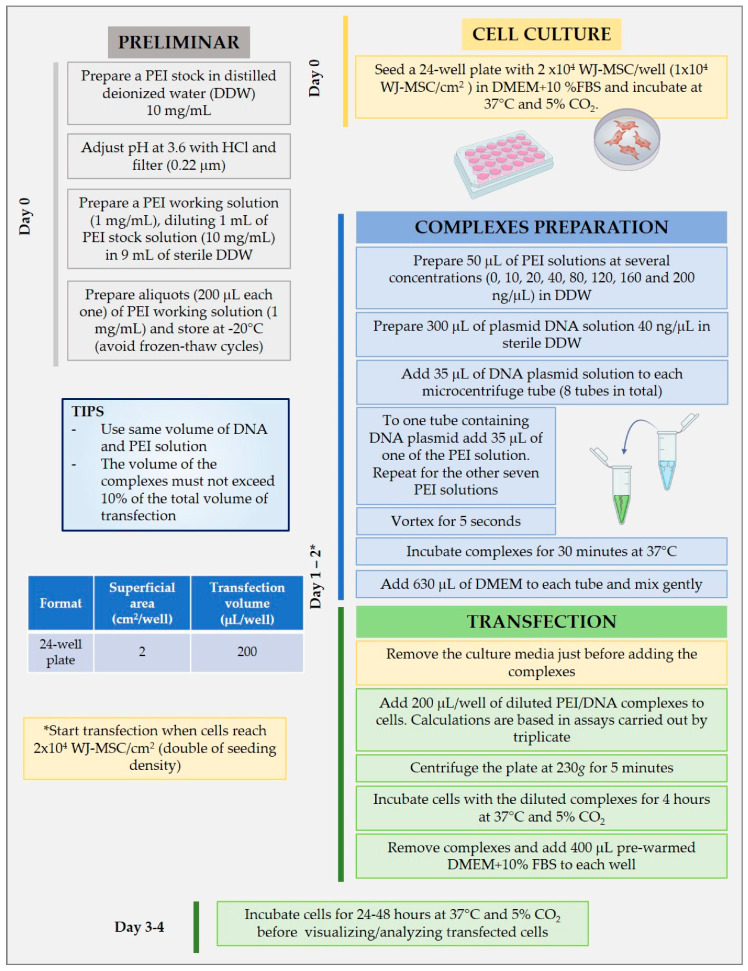
Detailed protocol used to transfect Wharton’s jelly-mesenchymal stem cells (WJ-MSC) with PEI in 24-well plates by triplicate.

MSC have been reported to inhibit the activation and proliferation of cells of the immune system. To assess the ability of WJ-MSC to maintain this ability after PEI transfection, an immunomodulation assay was performed, measuring the ability of transfected WJ-MSC to inhibit the proliferation of CD3+ T lymphocytes. Peripheral blood mononuclear cells (PBMC) were cultured in RPMI medium supplemented with 10% *v/v* FBS and used as a source of T lymphocytes. PBMC were previously activated with CD2, CD3 and CD28 monoclonal antibodies (130-091-441, T cell Activation/Expansion Kit, Miltenyi Biotec GmbH, Bergisch Gladbach, Germany). Initially, WJ-MSC were transfected following the procedure described in Figure 1, using an N/P ratio of 14. After 48 h of transfection, transfected cells were trypsinized, recovered by centrifugation, seeded into 24-well plates at 5 × 10^4^ cells per well, and cultured for 24 h. Resulting cells were then co-cultured with 5 × 10^5^ of activated PBMC (Act co-cul T) at 37 °C with 5% humidified CO_2_ for 5 days. Activated PBMC co-cultured with WJ-MSC untransfected (Act co-cul NT), activated PBMC (Act) and non-activated PBMC (NAct) were used as controls. After 5 days of culture, PBMC were recovered from wells, incubated at 4 °C for 30 min with antiCD3 antibody (300329, Pacific Blue™ anti-human CD3 Antibody, Clone HIT3a, Biolegend, San Diego, CA, USA), and then examined by flow cytometry.

### 2.10. Evaluation of Multilineage Differentiation Capacity of WJ-MSC

The multipotential capacity of the WJ-MSC after transfection with PEI at an N/P ratio of 14 (720 ng PEI/9 × 10^4^ cells) was examined by culturing transfected cells in secondary cultures using osteogenic and adipogenic differentiation media. To induce adipogenic differentiation, transfected cells were seeded into 24-well plates at 5 × 10^4^ cells per well and cultured until 60% confluency was achieved. At this point, medium was replaced with adipogenic induction medium (Stromal Pro Adipogenesis Differentiation Kit, Life Technologies, Carlsbad, CA, USA). The medium was changed every 3 days and after 14 days, cells were fixed in 4% paraformaldehyde (Sigma Aldrich, St Louis, MO, USA) before staining lipid vacuoles with Oil Red O (Sigma Aldrich, St Louis, MO, USA). For osteogenic differentiation, transfected cells were seeded similarly and exposed to osteogenic differentiation medium (Stromal Pro Osteogenesis Differentiation Kit, Life Technologies). Calcium deposition was analyzed using Alizarin red-S (Sigma Aldrich, St Louis, MO, USA) staining. For all controls (non-differentiation conditions), cells were kept in DMEM + 10% FBS under standard culture conditions. The procedure was repeated using non-transfected (NT) WJ-MSC. In all cases, observations were made using microscopic and photographic records.

### 2.11. Statistical Analysis

Statistical analyses were performed using Statgraphics Centurion 18 software (Statgraphics Technologies, Inc., The Plains, VA, USA). One-way ANOVA was used for analysis of variance with Tukey’s post hoc test for comparison between groups. Numerical and graphical results are displayed as mean ± standard deviation. Significance was accepted at a level of *p* < 0.05.

## 3. Results and Discussion

### 3.1. Mean Hydrodynamic Diameter and Zeta Potential Remain Constant with the Increase in PEI Concentration

**Figure 2:**
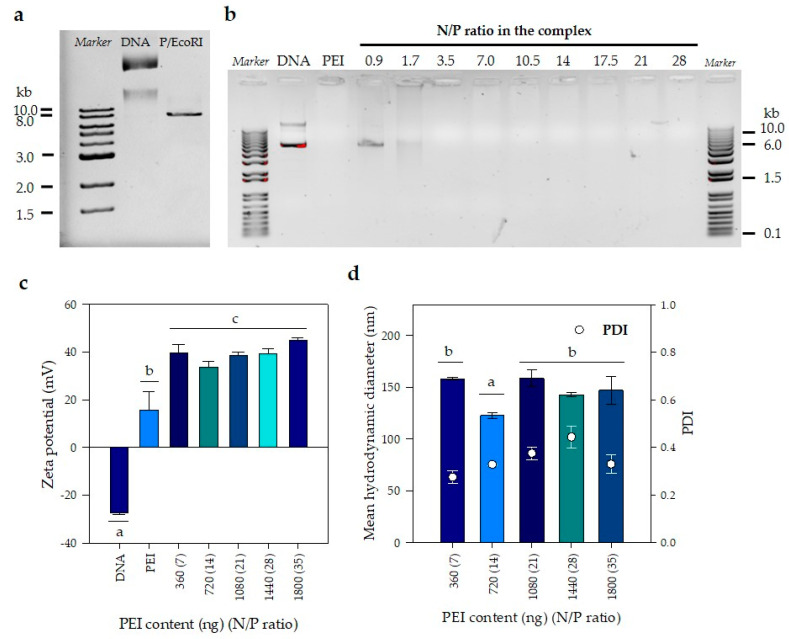
Evaluation of PEI/DNA complexes formation. (**a**) Gel retardation assay of the DNA plasmid to establish its size. (**b**) PEI/DNA complexes formation by gel retardation assay. A value of 400 ng of DNA plasmid was mixed with PEI solutions in distilled water (DW) at different concentrations to obtain complexes at several N/P ratios. N/P = 7 corresponds to 360 ng PEI. (**c**) Effect of N/P ratio on zeta potential. (**d**) Mean hydrodynamic diameter and polydispersity of the sample (PDI). ^(a–c)^ denotes significance (*n* = 3 technical replicates, *p* < 0.05) in comparison with all groups at the same time point. The error bars represent 1 SD.

DNA/PEI complexes used for transient transfection of WJ-MSC were characterized by electrophoresis and dynamic light scattering (DLS). First, in order to evaluate the performance of a gene delivery vector, it is necessary to establish the minimum N/P ratio for the effective complexation of a given amount of DNA. Furthermore, evaluation of transfection conditions to obtain an optimum N/P ratio at which transfection efficiency is maximized, is then required to reduce cell toxicity. For this purpose, the size of the plasmid used in the formation of complexes was evaluated by electrophoresis. Two bands were distinguished when plasmid was not digested in the second lane of Figure 2a; the same two bands were visible also in the second lane (DNA) of the gel shown in Figure 2a and very likely corresponded to the open-circular (oc) and covalently closed circular (ccc) DNA, which suffer from friction against the agarose matrix. The size of the linearized plasmid was resolved after digestion with EcoRI and corresponded to approximately 8 kb, as shown in Figure 2a. Once the plasmid size was established, complexes prepared at different PEI concentrations using the same amount (400 ng) of plasmid DNA at 40 ng/μL were evaluated by electrophoresis in agarose gel, where a delay shift of any given band suggested DNA complexation with PEI. At N/P ratios equal or lower than 1.7, we observed bands migrating in a similar pattern to the one observed for the plasmid alone, indicating that higher N/P ratios are required to allow the formation of PEI-DNA complexes (Figure 2b). At N/P ratios equal or greater than 3.5, DNA bands are not seen in the electrophoresis gel once there is enough PEI to complex with DNA, because the condensation of DNA with PEI either excludes or displaces ethidium bromide from intercalating with DNA; additionally, the cationic character of both ethidium bromide and PEI causes a repulsion between the two molecules, reducing or preventing the electrostatic interaction between DNA and ethidium bromide. As a result, the fluorescence will be reduced to the basal level of ethidium bromide in the aqueous solution of the gel and will no longer be detectable as a band in the corresponding lanes of the electrophoresis.

After establishing the conditions to form complexes, zeta potential and particle size were evaluated. As expected, due to the presence of phosphate groups on its surface, a negative zeta potential of approximately −27.0 ± 0.9 mV was obtained for a DNA stock solution at 400 ng/mL in DW; this value is slightly more negative than obtained by Mady et al. (2011) [35]. In contrast, the zeta potential of a PEI solution (33.3 µg/mL) in DW was positive, but oscillated between 6 and 26 mV, meaning the highest variability (coefficient of variation of 51%); such value is also slightly smaller than the reported by Mady et al. (2011) [35] and using the same analytic technique. The lowest N/P ratios evaluated were 1.7 and 3.5, and their zeta potential values were 17 ± 1 and 23 ± 2 mV, respectively. Under these conditions, it was not possible to measure the hydrodynamic sizes by DLS because samples were too polydisperse for the cumulative analysis used in the DLS technique. This behavior can be explained by comparing our results with those obtained by Mady et al. (2011) [35] who evaluated complex formation by measuring the relative fluorescence intensity emitted upon the addition of PEI to ethidium bromide (EB)-DNA complex (fluorescent light could be quenched by the addition of PEI that competes with EB to bind DNA). They reported that supernatants of complexes prepared at N/P values lower than 3.5, showed fluorescence suggest incomplete DNA condensation by PEI [35].

Figure 2c shows zeta potential measurements varied between 33 ± 3 and 45 ± 1 mV for N/P ratios between seven and 35, respectively. A Tukey multiple comparison test was performed to determine which zeta potential averages were significantly different from the others. Statistically significant differences were only found at a N/P ratio of 14 (confidence level of 95%). For the other N/P ratios, no differences were found (*p* < 0.05). Finally, the mean hydrodynamic diameter of the complexes was measured as a function of the N/P ratio. Figure 2d shows a slight but significant reduction in the mean hydrodynamic diameter when the N/P ratio was 14 (123 ± 3 nm) in comparison with the other four N/P ratios evaluated, which on average had a diameter of 152 ± 7 nm. It must be mentioned that the polydispersity index of those measurements ranged from 0.25 to 0.45, which means that in reality there was a wide distribution of particle sizes for the evaluated conditions; such distribution will be important when discussing the possible route of entrance of PEI/DNA complexes into the cell.

Han et al. (2009), indicated that, in the phase of formation of the PEI/DNA complexes, the outcome of the transported transgenes is largely defined [36]. Therefore, complex preparation is one of the critical steps to maximize the results of PEI-mediated protein expression processes [36]. Van Gaal et al. (2011) analyzed the different parameters varied in transfection assays with non-viral vectors, which are: a buffer used for complexation (water, NaCl, HEPES buffer), DNA dose, cell confluency, incubation period of complexes with cells and incubation medium (with or without serum) [37]. In addition to these parameters, Bono et al. (2020) also suggest an effect of the method of complex formation (by dripping, mixing with a pipette, homogenizing with vortex) and the relationship between the volume of the complex and the volume of culture medium. These operational aspects influence complex size, zeta potential, toxicity, and transfection efficiency [30].

Bono et al. (2020) compared the transfection efficiency of the complexes prepared in water with those prepared in 150 mM NaCl, using murine fibroblasts (L929 cell line) and 25 kDa linear PEI and found that the transfection efficiency of the complexes prepared in NaCl was increased 10 times compared to the complexes prepared in water [30]. They also observed that the particle size increased from 4.5 to nine times, going from particles with sizes between 100–200 nm when complexes were prepared in water, to others of 900 nm when prepared in NaCl. According to the authors, transfection efficiency is favored by larger particles, which settle faster than small ones, according to Stokes’ law that describes the movement of a sphere of diameter (*d*) in a gravitational field. According to Stokes’ equation (Equation (1)), the velocity of sedimentation (*v*) is given by the relation of the diameter of the sphere, the particle (*p*) and the medium (*L*) density, the gravitational force (*g*) and the viscosity of medium (*n*):

*v* = *d*^2^(*p* − *L*)*g*/18*n* (1)

In presence of a gravitational force, particles of higher density or larger size typically travel at a faster rate than particles less dense or smaller.

*d*_2_ = 900 nm, *v*_2_ = 810,000 *k*_2_, where *k*_2_ = (*p_2_ − L*)*g*/18*n*

*d*_1_ = 100 nm, *v*_1_ = 10,000 *k*_1_, where *k*_1_ = (*p_1_ − L*)*g*/18*n*

If we assume that the particle density remains constant, then *k*_2_ *= k*_1_, and the ratio (*r*) between the velocity of sedimentation will be *r* = *v*_2_*/v*_1_ = 81, thus, particle of 900 nm diameter, sediment 81 times faster than 100 nm diameter particle.

Meanwhile, Han et al. (2009) evaluated the effect of incubation time on the size of the complexes prepared in NaCl and found that the size of the complexes increases with the incubation time going from 750 nm (30 min) to 1750 nm (120 min). Han et al. (2009) postulated that the complexes must be stable enough not to dissociate in the cytoplasm but to dissociate in the nucleus for transfection and they also found that when the stability of the complexes is increased, the efficiency of endocytosis/phagocytosis is decreased [36]. Complex formation time was also evaluated. An increase from 10 to 120 min, reduced the transfection efficiency from 40 to 13%. They also found that the total apparent activity of both gene transcription and protein synthesis was negatively affected by complex formation time. That is, the longer the complex formation time, the greater the particle size, the less transfection efficiency [36].

Ogris et al. (1998) found that smaller particles (60–120 nm) which compacted too strongly to start transcription were formed in the absence of NaCl [38]. They found that intracellular complexes were dispersed mainly within 48 h after transfection and anticipated that high stability endocytosed complexes are likely to cause a delay in gene expression [38]. In our work, we prepared the PEI/DNA complexes in distilled water and found that the particle size (approximately 150 nm) and the zeta potential (greater than 30 mV) remained constant with the increase in PEI. Regarding the size of the particles, they are involved in the selection of the absorption pathways of non-viral genetic complexes, as well as the surface charge of the particle, the shape of the particle, the type of cell, and even the condition of the culture. PEI/DNA complexes with sizes less than 500 nm are mainly taken by CME (Clathrin-mediated endocytosis) and CvME (caveolae-mediated endocytosis), while PEI/DNA complexes with sizes greater than 500 nm are mainly internalized by the macropinocytosis pathway [39,40]. In COS-7 cells, von Gersdorff et al. (2006) found that transfection with PEI was mediated by the clathrin pathway. For HUH-7 transfection with linear PEI occurs mainly by the clathrin-dependent pathway, whereas for branched PEI transfection was mediated by CME and CvME. The same occurred for HeLa cells, where both pathways mediated transfection, with the caveolae pathway being the most efficient [41]. In CHO-1 and Hela, Hufnagel et al. (2009) found that for particles of the order of 500 nm, macropinocytosis played an important role in the absorption of the complexes [40].

We obtained particles of approximately 150 nm, however, the polydispersity values (PDI) varied between 0.3 and 0.5, which indicates that the size of the particles is highly heterogeneous and therefore, several pathways may be intervening in the entrance of the complexes to the cell. In summary, the size of the complexes can define the cell entry mechanism, as well as its disassembly and subsequent entry into the nucleus. Additionally, size is also associated with the stability of the complexes. Smaller sizes lead to the formation of more stable complexes that take time to disassemble, which could favor the transfection of cells such as WJ-MSC that have a lower proliferation rate than cell lines. Finally, in the case of WJ-MSC, it is necessary to assess whether the transfection is dependent on the cell cycle, since this dependence is a function of the cell type evaluated. Further studies should evaluate the dependence of WJ-MSC transfection on the cell cycle, since several authors have reported that the relation between transfection efficiency and cell cycle is cell dependent, and there are reports were authors there were found a correlation [42,43,44], while others have found that there is no dependency [36,43,45,46]. Additionally, it is necessary to evaluate the growth rate of WJ-MSC, as well as the effect of concentration on transfection efficiency and select conditions that optimize transfection efficiency—since PEI has been reported to be toxic [47,48,49,50,51,52,53,54,55]—and it is necessary to find the conditions that maximize transfection efficiency and at the same time have the least effect on cell viability.

### 3.2. Evaluation of Cell Toxicity Induced by PEI Complexes

**Figure 3:**
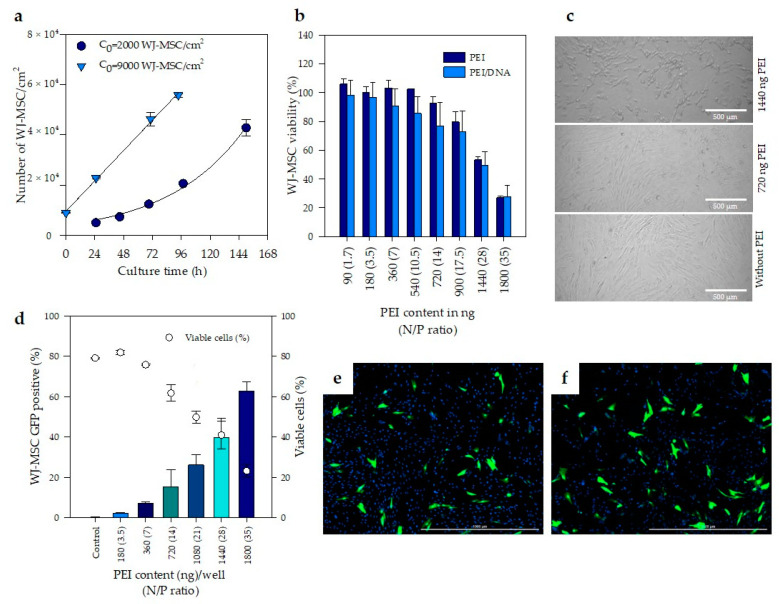
Transfection of WJ-MSC with PEI. (**a**) Effect of seeding density(C_0_) on the growth kinetics of WJ-MSC. (**b**) Cell viability of 4.5 × 10^4^ WJ-MSC/200 µL after 48 h of treatment with PEI and PEI/DNA complexes at different N/P ratios. (**c**) Bright-field photographs of non-treated WJ-MSC (without PEI) and treated with 720 and 1440 ng of PEI/well after 24 h. Scale bar: 500 µm. (**d**) Percentage of WJ-MSC expressing green fluorescent protein (GFP) by flow cytometry (*y*-axis, left) related with the percentage of viable cells in the same assay (*y*-axis, right). (**e**,**f**) Fluorescent microscope images of transfected WJ-MSC (48 h post-transfection). Scale bar: 1000 µm. (^a–c^) denotes significant differences (*n* = 3 biological replicates, *p* < 0.05) in comparison with all groups. The error bars represent 1 SD.

Cell type and source are some of the most critical factors impacting on transfection efficiency. The growth kinetics of WJ-MSC and the effect of seeding density (C_0_) were first assessed in order to standardize the transfection conditions of WJ-MSC. Results evidence that increasing 4.5 times the seeding density (from 2000 to 9000 cells/cm^2^) produces a decrease in the duplication rate at the first 19 h (Figure 3a). When the seeding concentration was 2000 cells/cm^2^ (approximately 5% confluence), exponential growth was observed with a doubling time of 40.5 h. However, at an initial cell concentration of 9000 cells/cm^2^, cell doublings were observed at 19 h, 33 and 50 h. Thus, increasing of cell seeding density reduce duplication rate, therefore, 9000 cells/cm^2^ was used for further assays. Likewise, the addition of PEI complexes into culture was fixed after 24 h of seeding, while the evaluation of transfection was conducted 24–48 h later to ensure that cells had divided at least once. In this way, at least one WJ-MSC generation is expected to be formed in the presence of PEI complexes, facilitating its entry into the cell, assuming that transfection of the MSC was mitosis-dependent.

Next, the effect of PEI and PEI/DNA complexes on the metabolic activity of WJ-MSC was evaluated using the resazurin assay technique. There were no statistically significant differences (*p* < 0.05) between the cell viability of WJ-MSC treated with PEI alone and PEI/DNA complexes (Figure 3b). This result suggests that the toxicity is caused both by the PEI that forms complexes with DNA, and by the PEI that remains free in the culture medium.

Additionally, the metabolic activity of WJ-MSC was reduced with the increase in PEI concentration. WJ-MSC treated with 1440 and 1800 ng of PEI in DW and PEI complexed with DNA (at 28 and 35 N/P ratios, respectively) reduced its viability to 50 and 70%, respectively, compared to untreated WJ-MSC. Figure 3c corresponds to bright field photographs of WJ-MSC used to evaluate metabolic activity of cells after treatment with PEI and PEI/DNA complexes (Figure 3b). Metabolic activity was related with cell viability. According to Figure 3b, cell viability of WJ-MSC treated with 720 and 1440 ng/well of PEI was 92.6 ± 4.6 and 53.3 ± 2.3%, respectively. Some morphological changes in the cells were observed with the increase in PEI content, accompanied by the appearance of small black dots, which probably correspond to cellular debris. In PEI-treated wells there are fewer cells attached to the plate, some cells begin to leave their elongated shapes and become circular.

Free PEI contributes to cell toxicity and—considering that these free molecules drive a faster and more efficient cellular internalization of polyplexes and contribute to the subsequent intracellular trafficking [56]—it is necessary to establish a balance between cell toxicity and transfection efficiency. In this regard, the percentage of WJ-MSC treated with PEI/DNA complexes expressing green fluorescent protein was quantified at day two post-transfection by flow cytometry (Figure 3d).

Figure 3d reported separately, the percentage of cells GFP positive (*y* axis, left) and the number of viable cells (*y* axis, right) as a function of PEI content. In this figure, it is possible to observe that maximum transfection obtained was 63%; however, at this condition cell viability was only 20%. Additionally, in Figure 3d it data on transfection efficiency (*y*) and PEI concentration (*x*) show a trend, closely described by a second order polynomic equation *y* = 1.3897 × 10^5^ – 0.5*x*^2^ + 8.5976 × 10^5^ – 0.3*x* + 8.3111 × 10^5^ – 0.1, R^2^ = 0.996. (Figure S1, Supplementary Materials). Additionally, when the cell viability data, measured by both flow cytometry and resazurin assay technique are analyzed; in both cases, the percentage of viable cells (*y* axis) declined following a lineal pattern starting from N/P ratio (*x*) of 3.5 (180 ng of PEI) up to N/P ratio of 35 (1800 ng of PEI), though there are slight variations depending on the used technique (Figures S2 and S3, Supplementary Materials).

In mathematical terms, it is possible to find an equation to maximize transfection by multiplying both equations (Table S1, Supplementary Materials), in order to obtain the number of viable GFP positive cells as a function of PEI content, then differentiating such function and equating to zero to find the maximum. Accordingly, the maximum number of transfected viable cells would be reached using between 1581 and 1606 ng of PEI; however, at this point cell viability would be near to 33–40%, as calculated from equations for flow cytometry and resazurin assay, respectively.

Despite its remarkable DNA condensation ability and transfecting efficiency, PEI also induces cytotoxicity in a concentration-dependent manner. Recent studies show the cytotoxic effects of PEI are caused by a series of different mechanisms, mainly disruption of the different membranes of the cell [57], including the outer cell membrane as well as internal membranes (i.e., endosome, mitochondria, endoplasmic reticulum (ER), Golgi apparatus and nuclear membrane) [47,48,49,50,51,52,53,54,55]. Other mechanisms include affected gene expression [58]. As a result, cytotoxicity of PEI remains a challenging issue, further complicated by the lack of clarity related to what PEI’s ultimate fate is, whether it is exocytosed or degraded (at least partially). This gap in knowledge is of clinical concern. However, at low enough concentrations, PEI has been cleared by the FDA for applications as an impregnant in the food-contact surface of regenerated cellulose sheets (21CFR177.1200); the FDA also approved, in march of 2015, the use of Adherus^®^ AutoSpray Dural Sealant, manufactured by Hyperbranch Medical Technology, Inc. (Durham, NC, USA), to prevent cerebrospinal fluid leakage in brain surgery; it contains a mixture of polyethylene glycol (PEG) ester solution and a polyethylenimine (PEI) solution which the body absorbs after 90 days [59]. Therefore, finding an appropriate concentration range to work with this polycationic molecule is essential.

Cautiously, we selected a PEI content of 720 ng (N/P ratio of 14) and used it as the “optimal” condition for further experiments; this “optimal” is relatively far from the calculated maximum, reducing the number of transfected viable cells by about 45%, but this lower content of PEI guarantees a much lower cytotoxicity (cell viability is larger than 80% as measured by resazurin).

Finally, in order to analyze cell morphology of transfected cells, fluorescent microscope images of WJ-MSC, stained with DAPI and transfected with complexes at N/P ratios of 14 and 21, were analyzed and are shown in Figure 3e,f, respectively. It is observed that the green fluorescent protein comprises the entire cell and that the morphology of the transfected cells is not affected by the transfection.

Our results are comparable with those obtained by Wang et al. (2011) and Tierney et al. (2013) who evaluated the transfection efficiency of PEI (branched, 25 kDa) in MSC from human bone marrow. Both authors obtained the same highest transfection efficiency of 25% under different conditions. On the one hand, Wang et al. (2011) used an N/P ratio of eight and a dose of 6 μg DNA/cm^2^, and calculated the N/P ratio based on the premise that only primary amines interact with DNA to form complexes and assuming that primary amines content in PEI is 25%. Therefore, in order to compare their results with ours, the N/P ratio reported by Wang et al. (2011) was corrected by multiplying it by four. Tierney et al. (2013), on the other hand, considered all the amines presented in the PEI and used a N/P ratio of seven and a dose of 1 μg DNA/cm^2^. Wang et al. (2011) and Tierney et al. (2013) used similar seeding densities (4180 to 5000 cells/cm^2^) and reported variations in cell viability after PEI transfection from 60 to 80%, respectively. In our assays, we use the double of the seeding density (9000 cells/cm^2^) and we reached transfection efficiencies near to 15% at a N/P of 14 and 0.4 μg of DNA with a cell viability of 80%. In comparation, we used 30 times less DNA than Wang et al. (2011), and five times less DNA than Tierney et al. (2013) to transfect the same number of cells [25,60].

Considering the work of Mady et al. (2011), where PEI added at a N/P value greater than 3.5 formed no complexes and remained free in solution, perhaps increasing the amount of DNA will also likely increase the number of particles and, therefore, the transfection efficiency. Taking into account that mean hydrodynamic diameter and zeta potential remained constant at N/P ratios from seven to 35 (Figure 2c,d), the variation in transfection efficiency is not likely associated with size and charge of the particles, but rather linked to free PEI that does not complex with DNA. According to Mady et al. (2011), when an N/P ratio of 3.5 is reached, the additional PEI added is not used in the formation of complexes, that is, it remains free in solution. This explains why transfection begins to be observed after a N/P ratio of 3.5. Transfection increased when N/P reached seven, peaked between 14 and 28, and then decreased when the N/P went up to 35, mainly due to toxicity [35]. Yue et al. (2011) showed that an excess of PEI in the solution improved the internalization of the complexes and contributed to the subsequent intracellular traffic [56]. Likewise, Hanzlíková et al. (2011) found that these free molecules are the ones that contribute the most to the toxicity during transfection [61]. These results suggest that there must be enough free PEI to promote transfection, but not that much to affect cell viability. Thus, optimal transfection of WJ-MSC is reached at a N/P ratio of 14.

In addition to PEI, various non-viral transfection systems have been employed for the genetic modification of MSC from different sources. The most studied MSC are those from bone marrow (hBM-MSC). Hoare et al. (2010) transfected hBM-MSC with commercially available cationic lipid, Lipofectamine (LF) 2000, and achieved transfection efficiencies between 20 and 40%, with the plasmid: lipid ratio increasing from five to 20, however the viability decreased from 80 to 50% [62].

To improve the viability and scalability of the agents used for transfection, the use of molecules such as glucocorticoids (GC) that are steroid hormones that regulate metabolic activity by binding the GC receptor and translocating it to the nucleus, where the receptor acts as a transcription factor to modulate gene expression, has been evaluated. Kelly et al. (2016) combined Lipofectamine-LTX (LF-LTX) with cortisol and dexamethasone and significantly improved hBM-MSC transfection, increasing transfection efficiency by up to three times and raising transgene expression by four to 15 times compared to non-glucocorticoid transfection [63].

Likewise, PEI has been used to improve transfection efficiency in MSC and reduce the toxicity of complexes. Saraf et al. (2008) modified branched polyethyleneimine (bPEI) with hyaluronic acid (HA), which is a natural ligand for CD 44, CD 54, and CD 168 receptors in hMSC, accomplishing an increased transfection efficiency of up to 4.6-fold relative to PEI alone and a concurrent reduction in toxicity by up to 80% [64]. Similarly, Delyagina et al. (2014) combined PEI/DNA complexes with biotin-streptavidin-adorned magnetic nanoparticles (MNP) to transfect hBM-MSC reaching efficiencies of 9.8 ± 7.5%, which are up to five times greater than those obtained with PEI alone [65].

These methodologies have been tested in MSC from different sources such as bone marrow and adipose tissue, among others. However, to date, few reports exist of the use of cationic polymers in the transfection of umbilical cord MSC. Bahadur et al. (2015), conjugated 1.2 kDa PEI with linoleic acid and hyaluronic acid and evaluated transfection in MSC of bone marrow (BM) and umbilical cord (UC) and used 25 kDa PEI as a transfection control. Although they reach transfection efficiencies of up to 40% in UC-MSC, the viability was reduced up to 60%, making it a methodology that can be improved [29]. Similar results were obtained with the functionalized particles used to transfect UC-MSC, where Wang et al. (2017) reached a transfection efficiency of 12% with PEI 25 kDa and 14% with the functionalized particles with a TAT peptide, even though with significant differences in cell viability, going from 70 to 90%, respectively [29].

Therefore, starting from the methodology presented here, it is possible to further improve transfection efficiencies using the variations proposed by other authors. While optimizing transfection, it is necessary to analyze the characteristic properties of MSC such as immunophenotype, immunomodulatory properties, and differentiation ability in vivo, considering that one of the goals of gene therapy is to improve or modulate these characteristics.

### 3.3. PEI Transfection Does Not Affect the Functional Properties of Wharton’s Jelly MSC

**Figure 4:**
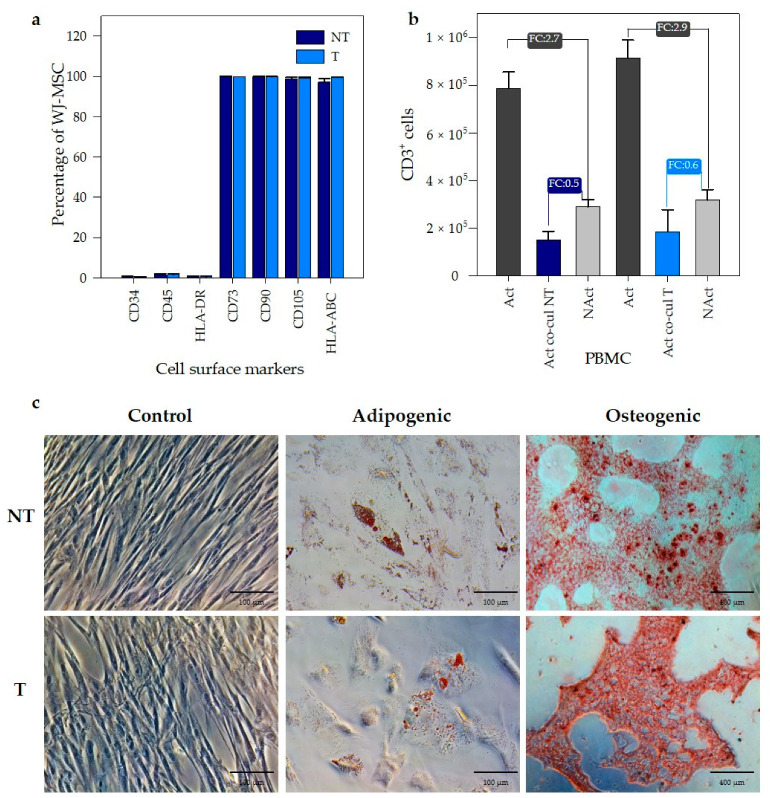
Transfection with PEI did not affect the functional properties of WJ-MSC. (**a**) Percentage of WJ-MSC expressing characteristic surface cell markers of mesenchymal stromal cells. (**b**) Immunomodulatory potency of WJ-MSC over peripheral blood mononuclear cells (PBMC). PBMC activated (Act) with CD2, CD3 and CD28 antibodies were co-cultured with non-transfected (Act co-cul NT) and transfected (Act co-cul T) WJ-MSC. Non-Activated (NAct) PBMC were used as controls. Data were expressed as absolute CD3+ cell counts for each condition. Fold change (FC) was expressed as the ratio between PBMC activated and non-activated. (*n* = 3 biological replicates, *p* < 0.05) in comparison with all groups. The error bars represent 1 SD. (**c**) Adipogenic (scale bar: 100 μm) and osteogenic (scale bar: 400 μm) differentiation of transfected (T) and non-transfected (NT) WJ-MSC with PEI at an N/P ratio of 14 (720 ng PEI).

Following the international criteria established by the Society for Cell and Gene Therapy (ISCT) to define multipotent mesenchymal stromal cells, we evaluated the effect of PEI transfection on WJ-MSC according to three identity factors: adherence to plastic in standard culture conditions, specific surface antigen (Ag) expression, multipotent differentiation potential (osteoblasts and adipocytes) demonstrated by in vitro cell culture staining [2] and one parameter of biological activity: the immunomodulatory properties. WJ-MSC transfected with PEI/DNA at an N/P ratio of 14 were evaluated. First, the presence of characteristic surface cell markers was analyzed by flow cytometry. WJ-MSC expressed no hematopoietic markers (CD34, CD45, and HLA-DR) in both transfected (T) and non-transfected (NT) cells (≤2%, Figure 4a). The presence of identity markers for MSC (CD73, CD90, and CD105) was positive (≥95%) in both transfected and non-transfected WJ-MSC.

The immunomodulatory capacity of WJ-MSC was evaluated as the ability to inhibit the proliferation of activated peripheral blood mononuclear cells (PBMC), measured as the number of CD3+ cells (specific marker for T lymphocytes). Figure 4b shows the number CD3+ cells for each evaluated condition. PBMC activated and co-cultured with transfected WJ-MSC (Act co-cul T) or non-transfected WJ-MSC (Act co-cul NT) were compared with PBMC non-activated (NAct). The ratio between them was expressed such as the fold change (FC) and was compared with the FC of PBMC activated (Act) and non-activated (NAct), used as the control. Figure 4b shows proliferation of activated PBMC in co-culture is inhibited by the presence of WJ-MSC. This inhibition in the growth of PBMCs is associated with the immunomodulatory capacity of WJ-MSCs. Given that the inhibition of activated PBMC proliferation occurred, both in the co-cultures of the transfected and non-transfected WJ-MSCs, these results suggest that the immunomodulatory capacity of the WJ-MSC was not affected by the transfection with PEI. Finally, we evaluated the impact of PEI-mediated transfection in the multipotent differentiation towards adipogenic and osteogenic lineages of WJ-MSC. The evaluation was performed in both transformed (T) and non-transformed (NT) cells. For adipogenic differentiation, we observed the formation of lipid vacuoles by staining with Oil Red in both experimental groups (Figure 4c), indicating the preservation of adipogenic potential after PEI transfection. Likewise, osteogenic lineage differentiation was evaluated using alizarin red-S. We observed the formation of calcium deposits in both T and NT WJ-MSC, as shown in Figure 4c. Then, we confirmed that MSC subjected to PEI transfection did not lost differentiation potential and immune-modulatory effects, suggesting PEI-based gene transfer as a safe method for MSC gene modification.

## 4. Conclusions

The overall aim of this study was to evaluate the efficacy of polyethylenimine (PEI) as a non-viral gene delivery system that can be optimized for gene therapy in human mesenchymal stromal cells isolated from umbilical cord Wharton’s jelly. Transfection efficiency achieved with our methodology gave a similar performance, using a lower quantity of DNA, compared to the results reported by other authors. Additionally, our results showed that WJ-MSC transfected with PEI retained their morphology, plastic adherence, immunophenotype, immunomodulatory function, and multi-lineage differentiation potential. Thus, PEI can be used as a transfection system in applications for therapeutic purposes. This improved methodology for transfection of WJ-MSC, based on PEI, has great potential for tissue engineering applications given the attractive differentiation capacity of MSC. Since engineered tissue constructs are made of three-dimensional structures, the next stage of the present work is to incorporate PEI-based transfection strategies to a three-dimensional scaffold that emulates the characteristics of the extracellular matrix. In this scenario, gene-activated three-dimensional scaffolds would serve as support for WJ-MSC, thus enabling gene transfer of inductive factors to favor osteogenic, adipogenic or chondrogenic differentiation to improve tissue regeneration.×

## Figures and Tables

**Table 1:** N/P ratio calculations to prepare polyethylenimine (PEI)/DNA complexes.

PEI (ng) without Correction	PEI (ng) Corrected by TN ^1^ Content	DNA (ng)	PEI/DNA Mass Ratio	Moles of N from PEI	Moles of P from DNA	N/P Ratio
100	90	400	0.2	2.1	1.2	1.7
200	180	400	0.5	4.2	1.2	3.5
400	360	400	0.9	8.4	1.2	7.0
800	720	400	1.8	16.7	1.2	14.0
1200	1080	400	2.7	25.1	1.2	20.9
1600	1440	400	3.6	33.5	1.2	27.9
2000	1800	400	4.5	41.9	1.2	34.9

^1^ TN: Total Nitrogen.

## Supplementary Materials

The following are available online at https://www.mdpi.com/1999-4923/12/9/896/s1, Figure S1: Second order polynomic patter of transfection by flow cytometry, Figure S2: Linear patter of cell viability by flow cytometry; Figure S3: Linear patter of cell viability by Resazurin assay technique, Table S1: Maximization of transfection and cell viability.
