# Supplementary material for: Efficient Non-Viral Gene Modification of Mesenchymal Stromal Cells from Umbilical Cord Wharton’s Jelly with Polyethylenimine
Source: Pharmaceutics. 2020 Sep 22;12(9):896. doi: 10.3390/pharmaceutics12090896 (PMC7559368; doi:10.3390/pharmaceutics12090896)
Supplement: Supplementary file 1 [file pharmaceutics-12-00896-s001.pdf]

pharmaceutics

Supplementary Materials: Efficient Non-Viral Gene Modification of Mesenchymal Stromal Cells from Umbilical Cord Wharton's Jelly with Polyethylenimine

Ana Isabel Ramos-Murillo, Elizabeth Rodríguez, Karl Beltrán, Cristian Ricaurte, Bernardo Camacho, Gustavo A. Salguero and Rubén Darío Godoy-Silva

Transfection efficiency (*y*) and PEI concentration (*x*) followed a trend, closely described by a second order polynomic equation y = 1.3897E-05x^2 + 8.5976E-03x + 8.3111E-01, R^2 =0. 996. (Figure S1)

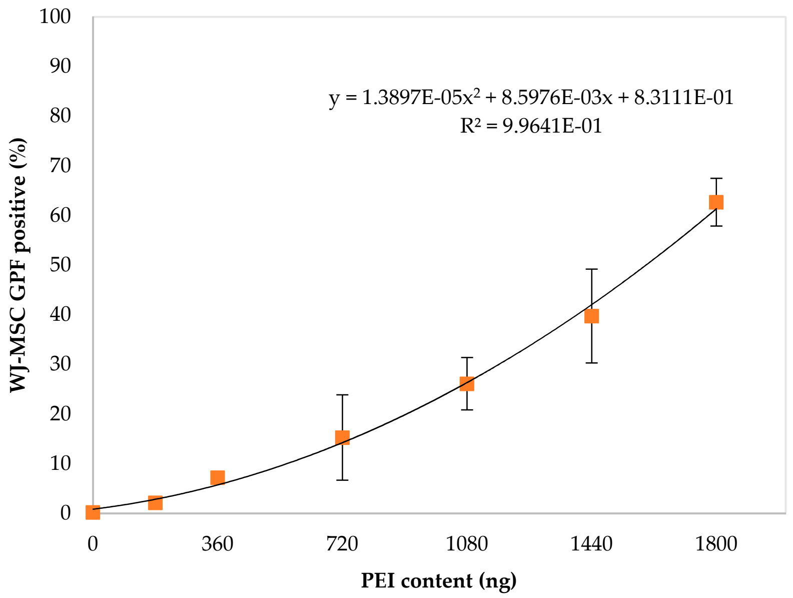

Figure S1. Second order polynomic patter of transfection by flow cytometry.

Analyzing cell viability data, measured by both flow cytometry and resazurin assay technique; in both cases, the percentage of viable cells (*y* axis) declined following a lineal pattern starting from N/P ratio (*x*) of 3.5 (180 ng of PEI) up to N/P ratio of 35 (1800 ng of PEI), though there are slight variations depending on the used technique, as shown in figures 2S and 3S:

pharmaceutics

MDPI

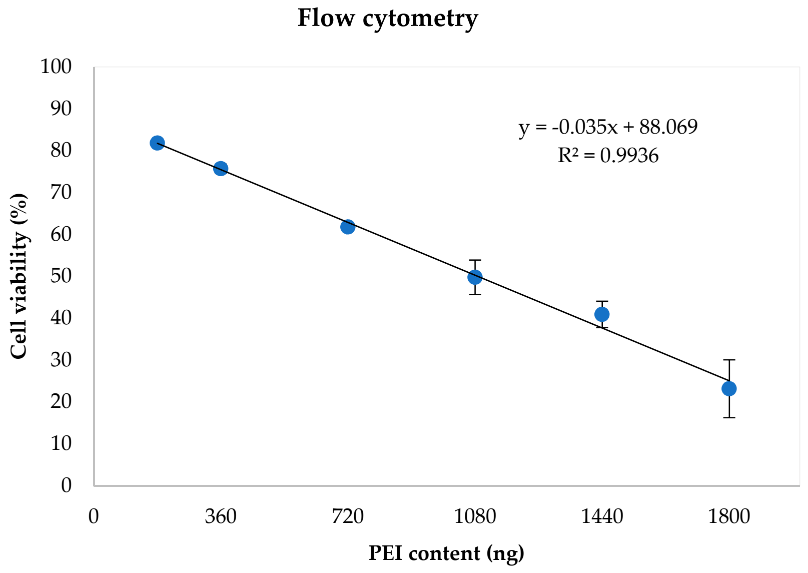

Figure S2. Linear patter of cell viability by flow cytometry.

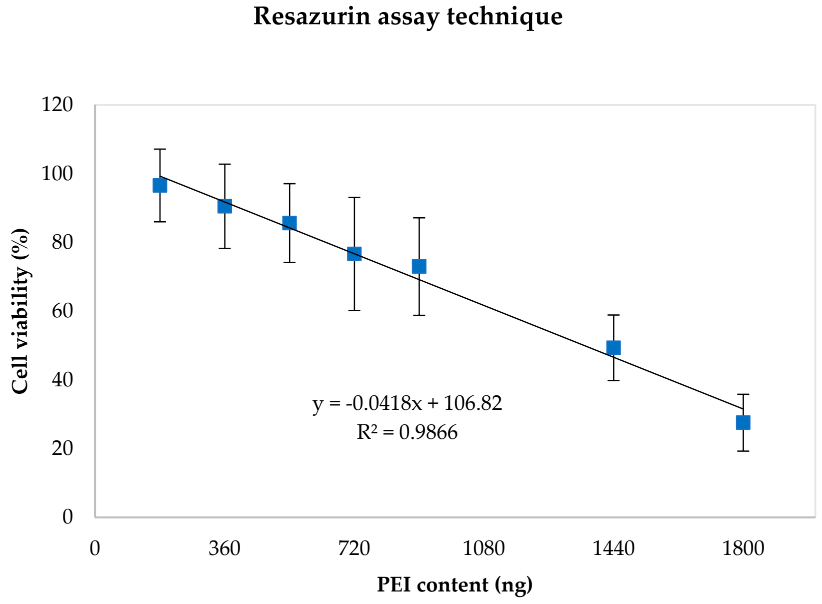

Figure S3. Linear patter of cell viability by Resazurin assay technique.

In mathematical terms, it is possible to find an equation to maximize transfection by multiplying both equations, in order to obtain the number of viable GFP positive cells as a function of PEI content, then differentiating such function and equating to zero to find the maximum, as shown in the table S1.

Table S1. Maximization of transfection and cell viability.

	**Transfection efficiency by Flow cytometry** $y = 1.3897E\text{-}05x^2 + 8.5976E\text{-}03x + 8.3111E\text{-}01$, $R^2 = 0.996$		Value of x (PEI content) when equation is equal to zero (x_{max})	

**Viability by flow cytometry** (y = -0.035x + 88.069, R^2 = 0.9936)	**Function to maximize:** $-4.86E-07x^3+9.23E-04x^2+7.28E-01x+7.32E+01$	**Derivative of the function to maximize:** $-1.46E-06x^2+1.85E-03x+0.73$	x_{max} = 1581 ng of PEI (the other value of x is negative, having no physical meaning)	Cell viability calculated at x_{max} = 33% Transfection efficiency calculated at x_{max} = 49%
**Viability by resazurin** (y = -0.0418x + 106.82, R^2 = 0.9866)	**Function to maximize:** $-5.81E-07x^3+1.12E-03x^2+8.84E-01x+8.88E+01$	**Derivative of the function to maximize:** $-1.74E-06x^2+2.25E-03x+0.88$	x_{max} = 1606 ng of PEI (the other value of x is negative, having no physical meaning)	Cell viability calculated at x_{max} = 40% Transfection efficiency calculated at x_{max} = 50%

Accordingly table 1, the maximum number of transfected viable cells would be reached using between 1581 and 1606 ng of PEI; however, at this point cell viability would be near to 33 – 40%, as calculated from equations for flow cytometry and resazurin assay, respectively.
